# Serine 13 of the human cytomegalovirus viral cyclin-dependent kinase UL97 is required for regulatory protein 14-3-3 binding and UL97 stability

**DOI:** 10.1016/j.jbc.2022.102513

**Published:** 2022-09-20

**Authors:** Satoko Iwahori, Angie C. Umaña, Robert F. Kalejta, Takayuki Murata

**Affiliations:** 1 Department of Virology and Parasitology, Fujita Health University School of Medicine, Toyoake, Aichi, Japan; 2 Institute for Molecular Virology and McArdle Laboratory for Cancer Research, University of Wisconsin-Madison, Madison, Wisconsin, USA

**Keywords:** cell cycle, phosphorylation, chaperone, proteomics, maribavir, kinase, 14-3-3 protein, FBS, fetal bovine serum, HCMV, human cytomegalovirus, IF, immunofluorescence, IP, immunoprecipitation, KD, kinase dead, MOI, multiplicity of infection, WB, Western blotting

## Abstract

The human cytomegalovirus (HCMV) UL97 protein is a conserved herpesvirus protein kinase (CHPK) and a viral cyclin-dependent kinase (v-CDK). However, mechanisms regulating its activity in the context of infection are unknown. Here, we identified several cellular regulatory 14-3-3 proteins as UL97-interacting partners that promote UL97 stability. Humans are known to encode seven isoforms of 14-3-3 proteins (β, ε, η, γ, σ, θ, and ζ) that bind phosphoserines or phosphothreonines to impact protein structure, stability, activity, and localization. Our proteomic analysis of UL97 identified 49 interacting partners, including 14-3-3 isoforms β, η, and γ. Furthermore, coimmunoprecipitation with Western blotting assays demonstrated that UL97 interaction with 14-3-3 isoforms β, ε, η, γ, and θ occurs in a kinase activity-dependent manner. Using mutational analysis, we determined the serine residue at amino acid 13 of UL97 is crucial for 14-3-3 interaction. We demonstrate UL97 S13A (serine to alanine substitution at residue 13) retains kinase activity but the mutant protein accumulated at lower levels than WT UL97. Finally, we show both laboratory (AD169) and clinical (TB40/E) strains of HCMV encoding UL97 S13A replicated with WT kinetics in fibroblasts but showed decreased UL97 accumulation. Taken together, we conclude that 14-3-3 proteins interact with and stabilize UL97 during HCMV infection.

The human cytomegalovirus (HCMV) UL97 protein functions as a viral cyclin-dependent kinase (v-CDK) (1, 2, 3). UL97 complements the growth of CDK-deficient yeast and shares phosphorylation substrates with cellular CDKs such as the retinoblastoma (Rb) tumor suppressor and the Rb family members p107 and p130 (1, 4, 5). Also, like the CDKs, UL97 phosphorylates and inactivates the deoxy-NTP (dNTP) hydrolase SAMHD1 (6, 7, 8) and Lamin A/C (9) proteins. Furthermore, UL97 phosphorylates viral proteins to support capsid nuclear egress and virion assembly (10, 11). Recombinant HCMVs lacking the UL97 gene or deficient for UL97 kinase activity grow poorly (12, 13).

Mechanisms that regulate the activity of cellular CDKs are well understood and include the phosphorylation of specific CDK residues, the interaction with cyclins and CDK inhibitors (CKIs), and associations with chaperone complexes (14). Despite the functional similarities between UL97 and cellular CDKs, our understanding of UL97 regulatory mechanisms is limited. UL97 is phosphorylated by CDK9/cyclin T1, but the significance of this event is unknown (15). Furthermore, UL97 associates with the cellular cyclins A2, B1, H, and T1, but whether these associations affect kinase activity or substrate specificity has not been established (15, 16, 17). While the CDK inhibitor p21 does not efficiently inhibit UL97-mediated phosphorylation compared with cellular CDKs (1), other CKIs have not been tested for their ability to regulate UL97 kinase activity. Similarly, if and how the other v-CDKs (EBV BGLF4; HHV-6a, HHV-6b, and HHV-7, U69; KSHV ORF36) (2) are regulated is not well established.

Compared to the other v-CDKs, UL97 contains an N-terminal extension predicted to be largely disordered (18). This N-terminal domain contains multiple autophosphorylation sites (19, 20, 21), including Ser-2, Ser-3, Ser-11, Ser-13, Thr-16, Thr-18, Ser-133, Thr-134, and Thr-177. While the N-terminal domain is dispensable for *in vitro* kinase activity (19), it promotes UL97 nuclear localization and overall viral fitness (22). More insight into the functions of this unique N-terminal extension of UL97 is required to understand its role during infection.

To understand the roles of UL97 and its N terminus, we, as well as others (3, 11, 16), have used molecular proteomics to identify UL97 interacting proteins. Here, we report the capture and identification of 49 candidate UL97 binding proteins. Among the novel interactors we found were a subset of the 14-3-3 proteins. Humans encode 7 isoforms of 14-3-3 proteins (β, ε, η, γ, σ, θ, and ζ) that form either homodimers or heterodimers depending on the isoforms (23). 14-3-3 proteins bind to phosphorylated Ser and/or Thr residues on client proteins and can affect their structure, stability, activity, and localization (23, 24). Proteins involved in cell cycle progression and the DNA damage response are prominent 14-3-3 clients (25, 26, 27, 28, 29).

We show here that UL97 binds to five different 14-3-3 proteins (β, ε, η, γ, and θ) dependent upon the Ser-13 residue of UL97, an autophosphorylation site found in its unique N-terminal extension. Interaction with 14-3-3 proteins increases UL97 stability but did not substantially contribute to viral fitness in the *in vitro* productive replication assays commonly employed to study HCMV. Our work identifies a unique function for the N terminus of UL97 and highlights 14-3-3 proteins as additional cellular regulators of HCMV.

## Results

### 14-3-3 **β**, **ε**, **η**, **γ,** and **θ** interact with HCMV UL97

To discover potential substrates and regulatory factors, we identified UL97-associated proteins from the HEK-293T cells commonly used for interactome studies (30, 31). Carboxy-terminally Halo-tagged UL97 or an unanchored Halo peptide were expressed by transient transfection in HEK-293T cells, and Halo-containing or Halo-associated proteins were collected from lysates with HaloLink beads and identified by mass spectrometry (LC-MS/MS). Two independent biological replicates were analyzed. Forty-nine UL97-interacting proteins (Table 1 and Supporting information) were detected in each of the two biological replicates but not in either of the two Halo-only negative control pull downs. Of these 49, 16 (AIFM1, CCT3, CCT7, CDC20, CDC37, CDK1, CDK2, CCNA2, CCNB1, DNAJA1, DNAJA2, EIF4A1, HSPA6, NUMA1, TRIM28, and VCP) have been detected in previous UL97 interactome analysis (3, 11, 15, 16, 17) and 5 (CCNB1, MARK2, PGRMC1, STUB1, and TRIM28) have been previously identified as UL97 substrates (20, 32). Although not all known interacting proteins and substrates were detected, our ability to identify multiple known UL97-interacting proteins and substrates suggests that the novel proteins we identified are promising candidates for true UL97-binding partners or targets.

**Table 1:** List of UL97 interacting proteins

Accession #	Protein name (49)	Ave. Ctrl^a^	Ave. UL97^a^	Previously identified as UL97 binding partner or kinase substrate
P17066	HSP76_Heat shock 70 kDa protein 6 GN = HSPA6	0	21.8795	Binding partner (11)^b^
P61981	1433G_14-3-3 protein gamma GN = YWHAG	0	12.164	
P31946	1433B_14-3-3 protein beta/alpha GN = YWHAB	0	11.104	
Q16543	CDC37_Hsp90 co-chaperone Cdc37 GN = CDC37	0	11.104	Binding partner (16)
Q13263	TIF1B_Transcription intermediary factor 1-beta GN = TRIM28	0	10.8147	Kinase substrate (20); Binding partner (16)
Q04917	1433F_14-3-3 protein eta GN = YWHAH	0	8.6553	
Q92598	HS105_Heat shock protein 105 kDa GN = HSPH1	0	5.319	
P31689	DNJA1_DnaJ homolog subfamily A member 1 GN = DNAJA1	0	5.18545	Binding partner (16)
P06493	CDK1_Cell division protein kinase 1 GN = CDK1	0	4.85785	Binding partner (16)
P14635	CCNB1_G2/mitotic-specific cyclin-B1 GN=CCNB1	0	4.5691	Binding partner (3, 15, 16, 17); Kinase substrate (32)
Q99832	TCPH_T-complex protein 1 subunit eta GN = CCT7	0	4.04745	Binding partner (16)
P49368	TCPG_T-complex protein 1 subunit gamma GN = CCT3	0	4.04745	Binding partner (16)
P27448	MARK3_MAP/microtubule affinity-regulating kinase 3 GN=MARK3	0	3.43105	
P49411	EFTU_Elongation factor Tu, mitochondrial GN=TUFM	0	3.1311	
P24941	CDK2_Cell division protein kinase 2 GN = CDK2	0	2.6983	Binding partner (16)
P26641	EF1G_Elongation factor 1-gamma GN = EEF1G	0	2.4095	
Q12931	TRAP1_Heat shock protein 75 kDa, mitochondrial GN = TRAP1	0	2.1207	
O60884	DNJA2_DnaJ homolog subfamily A member 2 GN = DNAJA2	0	2.0819	Binding partner (16)
Q7KZI7	MARK2_Serine/threonine-protein kinase MARK2 GN = MARK2	0	2.0819	Kinase substrate (20)
Q92841	DDX17_Probable ATP-dependent RNA helicase DDX17 GN = DDX17	0	2.04315	
Q12834	CDC20_Cell division cycle protein 20 homolog GN = CDC20	0	2.00434	Binding partner (3, 16)
P55072	TERA_Transitional endoplasmic reticulum ATPase GN = VCP	0	1.71555	Binding partner (16)
Q15084	PDIA6_Protein disulfide-isomerase A6 GN = PDIA6	0	1.67674	
O95831	AIFM1_Apoptosis-inducing factor 1, mitochondrial GN = AIFM1	0	1.67674	Binding partner (16)
Q14697	GANAB_Neutral alpha-glucosidase AB GN = GANAB	0	1.426735	
P62306	RUXF_Small nuclear ribonucleoprotein F GN = SNRPF	0	1.38795	
P07737	PROF1_Profilin-1 GN = PFN1	0	1.38795	
P18669	PGAM1_Phosphoglycerate mutase 1 GN = PGAM1	0	1.38795	
Q9UNE7	CHIP_E3 ubiquitin-protein ligase CHIP GN = STUB1	0	1.38795	Kinase substrate (32)
Q14980	NUMA1_Nuclear mitotic apparatus protein 1 GN = NUMA1	0	1.34914	Binding partner (16)
P78406	RAE1L_mRNA export factor GN = RAE1	0	1.34914	
Q9HCN8	SDF2L_Stromal cell-derived factor 2-like protein 1 GN = SDF2L1	0	1.060385	
P05141	ADT2_ADP/ATP translocase 2 GN = SLC25A5	0	1.060385	
P35613	BASI_Basigin GN = BSG	0	1.060385	
P60842	IF4A1_Eukaryotic initiation factor 4A-I GN = EIF4A1	0	1.060385	Binding partner (16)
Q15233	NONO_Non-POU domain-containing octamer-binding protein GN = NONO	0	1.060385	
O43175	SERA_D-3-phosphoglycerate dehydrogenase GN = PHGDH	0	1.060385	
P31040	DHSA_Succinate dehydrogenase [ubiquinone] flavoprotein subunit, mitochondrial GN = SDHA	0	1.060385	
P60660	MYL6_Myosin light polypeptide 6 GN = MYL6	0	1.02154	
P30086	PEBP1_Phosphatidylethanolamine-binding protein 1 GN = PEBP1	0	1.02154	
O00264	PGRC1_Membrane-associated progesterone receptor component 1 GN = PGRMC1	0	1.02154	Kinase substrate (20)
Q93008	USP9X_Probable ubiquitin carboxyl-terminal hydrolase FAF-X GN = USP9X	0	1.02154	
P31942	HNRH3_Heterogeneous nuclear ribonucleoprotein H3 GN=HNRNPH3	0	1.02154	
P00558	PGK1_Phosphoglycerate kinase 1 GN = PGK1	0	1.02154	
P20248	CCNA2_Cyclin-A2 GN = CCNA2	0	1.02154	Binding partner (16, 17)
P30101	PDIA3_Protein disulfide-isomerase A3 GN = PDIA3	0	1.02154	
P30153	2AAA_Serine/threonine-protein phosphatase 2A 65 kDa regulatory subunit A alpha isoform GN = PPP2R1A	0	1.02154	
Q9UBS4	DJB11_DnaJ homolog subfamily B member 11 GN = DNAJB11	0	0.693975	
Q99615	DNJC7_DnaJ homolog subfamily C member 7 GN = DNAJC7	0	0.693975	

a Average of quantitative value normalized total spectra for control (HALO only) or UL97 in duplicates.

b Reference number.

**Figure 1:**
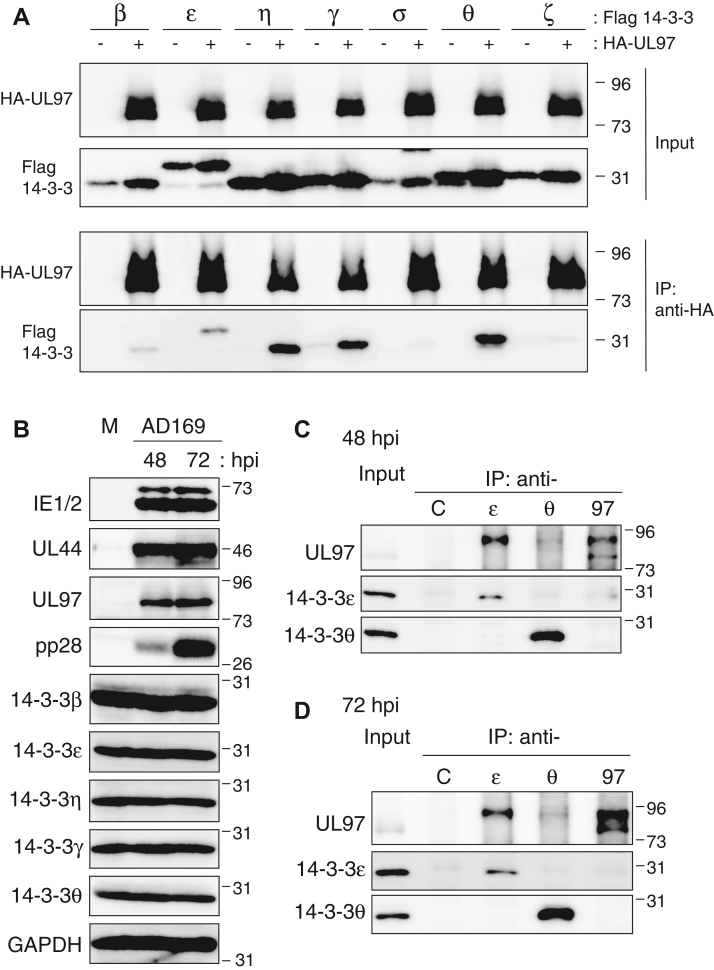
**HCMV UL97 interacts with 14-3-3 β, ε, η, γ, and θ.***A*, U-2 OS cells were transfected with an expression vector for HA-tagged UL97 or an empty vector (−) together with expression vectors for one of each of the seven isoforms of the Flag-tagged 14-3-3 proteins. After 48 h, lysates (Input) were harvested, subjected to immunoprecipitation (IP) with anti-HA antibody (IP: anti-HA) and analyzed by Western blot with the indicated antibodies. *B*, hTERT-immortalized human foreskin fibroblasts (HFF2/T) were mock infected (M) or infected with HCMV (strain AD169) at an MOI of 3 in the presence of 10% FBS. Lysates prepared at the indicated hours post-infection (hpi) were analyzed by Western blotting with the indicated antibodies. *C*, lysates (Input) from panel (*B*) (48 hpi) were subjected to IP with normal rabbit IgG as a control and anti-14-3-3 ε, θ, and UL97 antibodies (IP: anti-C, ε, θ, 97) and analyzed by Western blot with the indicated antibodies. *D*, IPs as in panel (*C*) from 72 hpi lysates. For all panels, representative images from one of three independent biological replicates are shown. FBS, fetal bovine serum; MOI, multiplicity of infection.

Our interactome contained three 14-3-3 proteins, β, η, and γ. Because UL97 interacted with multiple 14-3-3 proteins and because very little is known about 14-3-3 regulation of HCMV infection (33), we further analyzed the physical and functional interaction between UL97 and the cellular 14-3-3 proteins. We screened all seven human 14-3-3 isoforms (β, ε, η, γ, σ, θ, and ζ) with transient transfection coimmunoprecipitation (IP) assays for their ability to bind HA-tagged UL97. We found that the three isoforms detected in the Halo-UL97 pulldown (β, η, and γ), as well as two others (ε and θ) interacted with HA-UL97 (Fig. 1*A*). Any putative binding of UL97 to 14-3-3 σ or ζ was below the limit of detection of this assay.

The steady state levels of the UL97-interacting 14-3-3 proteins did not change during HCMV infection (Fig. 1*B*). Of the five isoforms that interact with UL97, we found antibodies for two (ε and θ) that worked for IP and used them to examine the interaction between UL97 and 14-3-3 protein in HCMV-infected cells. Antibodies for 14-3-3 ε or θ coimmunoprecipitated UL97 at both 48 hpi (Fig. 1*C*) and 72 hpi (Fig. 1*D*). Reciprocal pull downs with UL97 antibodies failed to consistently coimmunoprecipitate the 14-3-3 proteins. Nevertheless, based on the unbiased proteomics, transfection, and infection experiments, we conclude that HCMV UL97 interacts with cellular 14-3-3 proteins β, ε, η, γ, and θ.

### 14-3-3 isoform-specific residues promote interaction with UL97

**Figure 2:**
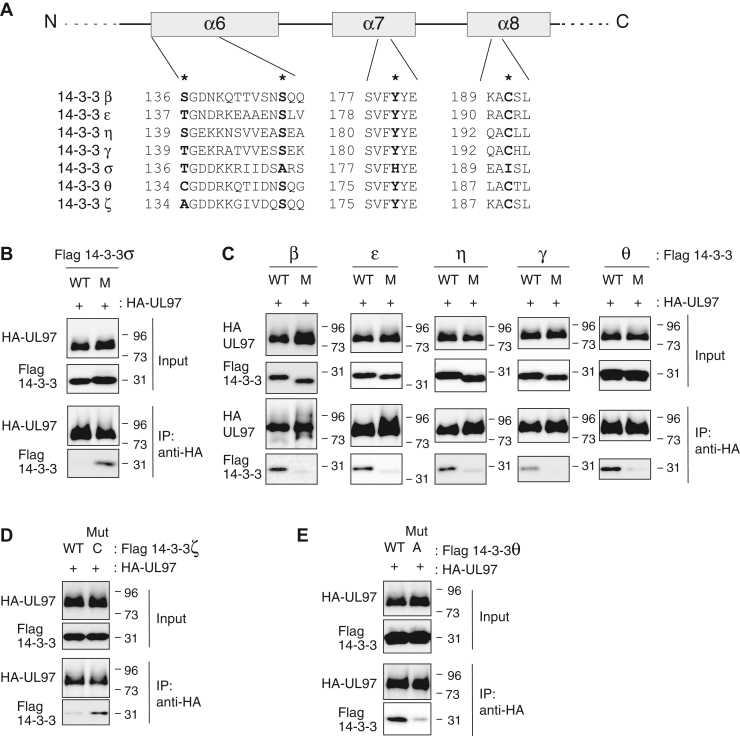
**Isoform-specific residues of the 14-3-3 proteins promote interaction with UL97.***A*, schematic diagram of the sixth, seventh, and eighth α helices from seven 14-3-3 isoforms. The asterisks (∗) indicate the amino acid residues tested for binding specificity with UL97. *B*, U-2 OS cells were transfected with an expression vector for HA-tagged UL97 together with expression vectors for either WT 14-3-3σ or a mutant (M) with three amino acid changes (A147S/H180Y/I191C). Lysates (Input) were subjected to immunoprecipitation (IP) with anti-HA antibody (IP: anti-HA) and analyzed by Western blot with the indicated antibodies. *C*, IPs as in panel (*B*) with triple amino acid mutants (M) of 14-3-3β (S147A/Y180H/C191I), 14-3-3ε (S148A/Y181H/C192I), 14-3-3η (S150A/Y183H/C194I), 14-3-3γ (S150A/Y183H/C194I), or 14-3-3θ (S145A/Y178H/C189I). More expression vector for mutated 14-3-3β (1 μg) than WT 14-3-3β (0.4 μg) was used to obtain similar expression levels. *D*, IPs as in panel (*B*) with a 14-3-3ζ (A134C) mutant (Mut C). *E*, IPs as in panel (*B*) with a 14-3-3θ (C134A) mutant (Mut A). For all panels, representative images from one of two independent biological replicates are shown.

Similar to UL97, the glutamate ionotropic receptor N-methyl-D-aspartate type subunit 2C (GRIN2C, also called GluN2C), a subunit of the N-methyl-D-aspartate receptor, binds to several 14-3-3 isoforms but not the σ isoform (34). Isoform-specific determinants for GluN2C binding to 14-3-3 proteins have been mapped to σ isoform-specific residues that disrupt binding (alanine-147 in α helix 6, histidine-180 in α helix 7, and isoleucine-191 in α helix 8) (Fig. 2*A*). The corresponding residues in each of the other 14-3-3 isoforms are different from the σ isoform but conserved with each other (serine instead of alanine in α helix 6, tyrosine instead of histidine in α helix 7, and cysteine instead of isoleucine in α helix 8) and promote GluN2C binding (34).

We found the residues that control 14-3-3 isoform-specific binding to GluN2C also control isoform-specific binding to UL97. For example, while the σ isoform fails to interact with UL97, a triple mutant converting the three key σ residues listed previously to the corresponding residues of the other 14-3-3 isoforms (A147S/H180Y/I191C) was able to bind UL97 (Fig. 2*B*). Furthermore, 14-3-3 isoforms β, ε, η, γ, and θ with these amino acids mutated to the σ isoform-specific residues (*e.g*., for the β isoform: S147A/Y180H/C191I) showed reduced interaction with UL97 (Fig. 2*C*). While these three residues dictate isoform-specific binding, they cannot do so exclusively, as the ζ isoform shares the binding promoting residues (Fig. 2*A*) but still fails to bind UL97. Interestingly, α helix 6 of the ζ isoform initiates with a nonpolar amino acid (alanine-134) while all the other isoforms contain a polar, noncharged residue at this position (cysteine, serine, or threonine) (Fig. 2*A*) (35). A mutant form of the ζ isoform with the nonpolar alanine replaced with a polar, noncharged cysteine (A134C) showed improved binding (Fig. 2*D*), whereas a reciprocal θ isoform mutant (C134A) showed reduced binding (Fig. 2*E*). In total, we conclude that UL97 utilizes isoform-specific residues in the sixth, seventh, and eighth α helices of 14-3-3 proteins for binding specificity.

### UL97 Serine-13 is required for interaction with 14-3-3 proteins during transient transfections

**Figure 3:**
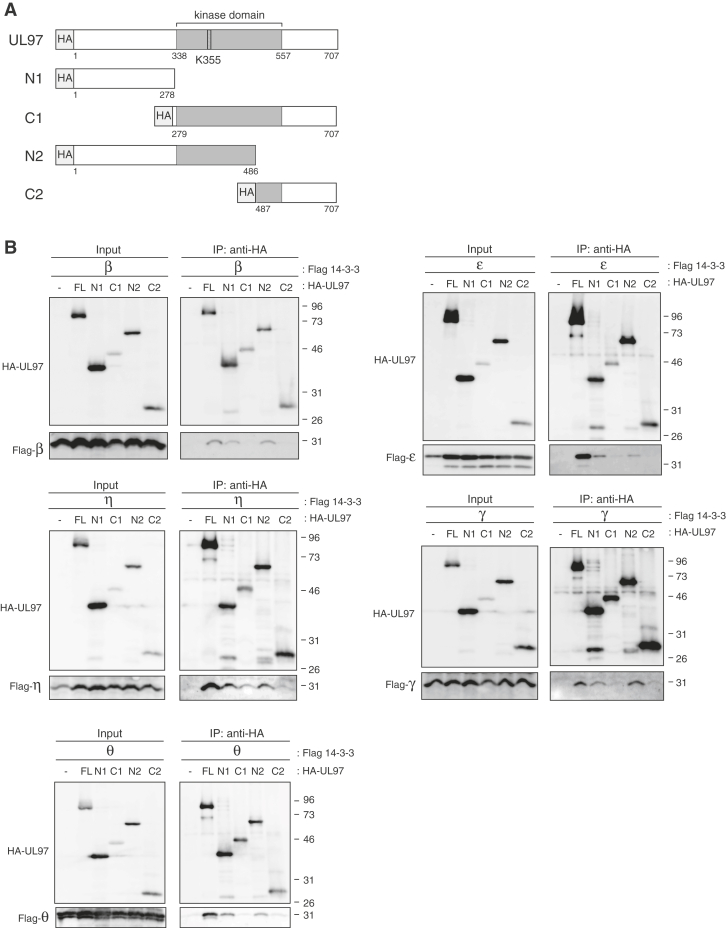
**The N-terminal 278 amino acids of UL97 contain the minimal 14-3-3 binding domain.***A*, schematic representation of the truncated forms of UL97 used in this study. *B*, U-2 OS cells were transfected with an expression vector for the indicated HA-tagged UL97 fragment (FL, full length; N1, C1, N2, C2, truncated forms) or an empty vector (−) together with expression vectors for the indicated Flag-tagged 14-3-3 isoform. After 48 h, lysates (Input) were harvested, subjected to immunoprecipitation with anti-HA antibody (IP: anti-HA), and analyzed by Western blot with the indicated antibodies. For all panels, representative images from one of three independent biological replicates are shown.

We tested epitope-tagged UL97 fragments (Fig. 3*A*) in transient transfection-coIP assays to identify the region of UL97 responsible for 14-3-3 binding. For each of the five UL97-interacting 14-3-3 isoforms, interaction was detected with UL97 fragments for the amino terminus N1 (amino acids (a.a.) 1 to 278) and N2 (a.a. 1–486) but not the carboxy terminus C1 (a.a. 279–707) and C2 (a.a. 487–707) (Fig. 3*B*). We conclude that the N-terminal 278 amino acids of UL97 contain the minimal 14-3-3 binding domain.

**Figure 4:**
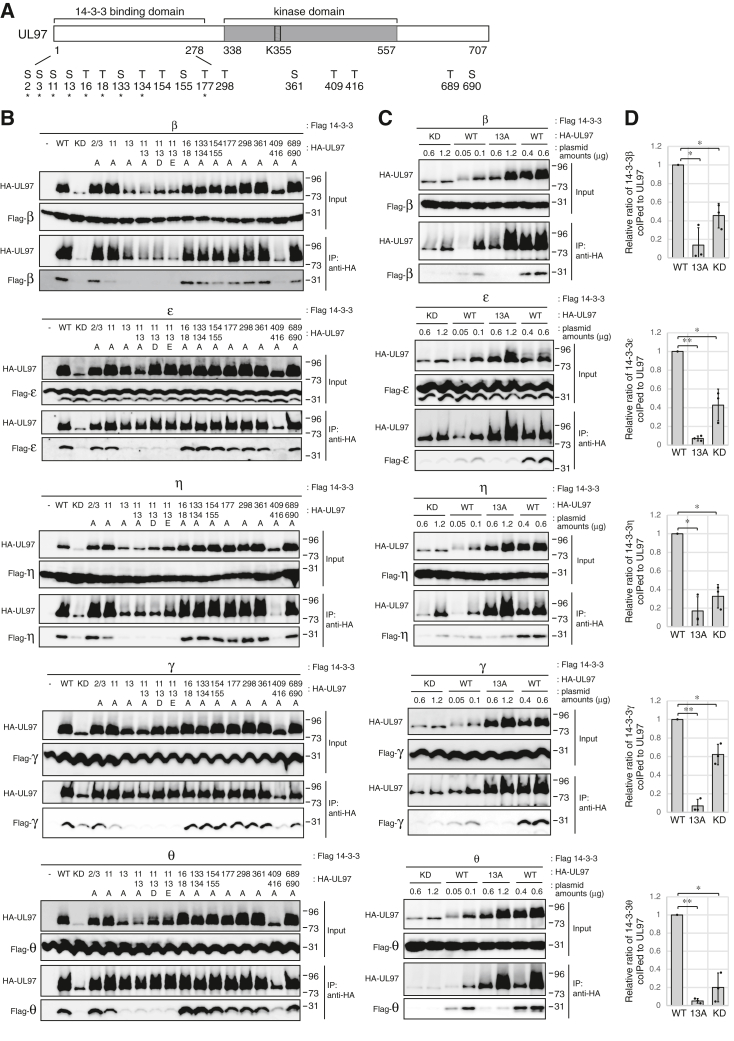
**UL97 Serine-13 is required for interaction with 14-3-3 proteins during transient transfections.***A*, the 14-3-3 binding domain and putative responsible residue(s) on UL97. The asterisks (∗) indicate previously identified autophosphorylation sites. *B*, U-2 OS cells were transfected with an expression vector for the indicated HA-tagged UL97 mutant (see panel *A* and Table 2, WT; KD, kinase dead (K355Q)) or an empty vector (−) together with expression vectors for the indicated Flag-tagged 14-3-3 isoform. After 48 h, lysates (Input) were harvested, subjected to immunoprecipitation with anti-HA antibody (IP: anti-HA) and analyzed by Western blot with the indicated antibodies. *C*, U-2 OS cells were transfected with a constant amount (1 μg) of an expression plasmid for the indicated Flag-tagged 14-3-3 isoform together with the indicated amounts (μg) of expression plasmids for WT UL97 or the indicated mutant. Immunoprecipitations were performed and analyzed as in panel (*B*). For all panels, representative images from one of two or three independent biological replicates are shown. *D*, quantification of the levels of bound 14-3-3 isoform normalized to immunoprecipitated WT UL97 or UL97 mutants from panel (*C*). WT UL97 (transfection with 0.6 μg of expression vector) and UL97 mutants (transfection with 1.2 μg for expression vectors of UL97-S13A or KD UL97) were compared. Values are presented relative to the value with WT UL97 (set at 1). Error bars denote the SD of three biological replicates. ∗*p* < 0.05; ∗∗*p* < 0.01.

14-3-3 proteins often bind to phosphorylated Ser/Thr residues on their clients (23). We found that a kinase dead (KD) allele of UL97 (K355Q) (catalytic lysine 355 converted to glutamine; Fig. 4*A*) accumulated to lower levels and showed reduced or eliminated binding to all five 14-3-3 proteins tested (Fig. 4*B*). The lack of autophosphorylation of UL97-KD can be observed by its enhanced electrophoretic mobility as compared to WT UL97. Dose-response experiments (Fig. 4*C*) and their quantitation (Fig. 4*D*) showed that UL97-KD, when accumulated to similar levels as UL97-WT, failed to associate with or less efficiently associated with all 14-3-3 proteins tested (Fig. 4, *C* and *D*). While incapable of autophosphorylation, UL97 N-terminal fragments still associate with 14-3-3 proteins, although far less efficiently than the full-length protein (Fig. 3), perhaps because they are inefficiently phosphorylated by cellular kinases (15). We conclude that the kinase activity of UL97, perhaps for autophosphorylation, is required for efficient 14-3-3 binding.

All nine previously identified (19) autophosphorylation sites (Table 2 and Fig. 4*A*) are within the newly identified 14-3-3 binding domain of UL97 (Fig. 3). Furthermore, we found 10 potential 14-3-3 binding sites in UL97 (Table 2) predicted by at least one of three independent algorithms (36) but only two of which were autophosphorylation sites (Ser-11 and Ser-13). In total, there are 17 serine or threonine residues in UL97 that could potentially mediate 14-3-3 binding (Table 2 and Fig. 4*A*). Of these, only Ser-13 is contained within the previously mapped 14-3-3 binding domain, autophosphorylated, and predicted by all three algorithms as a potential 14-3-3 binding site (Table 2).

**Table 2:** Autophosphorylation sites and predicted 14-3-3 binding sites on UL97

Ser/Thr	Surrounding sequence^a^	Autophosphorylation^b^	ANN^c^	PSSM^d^	SVM^e^
Ser-2	M**S∗**SALRS	+	−	−	−
Ser-3	MS**S∗**ALRSR	+	−	−	−
Ser-11	RSRAR**S∗**ASLGT	+	+	+	−
Ser-13	RARSAS∗LGTTT	+	+	+	+
Thr-16	SASLG**T∗**TTQGW	+	−	−	−
Thr-18	SLGTT**T∗**QGWDP	+	−	−	−
Ser-133	RPVVP**S∗**TSSRG	+	−	−	−
Thr-134	PVVPS**T∗**SSRGS	+	−	−	−
Thr-154	LRCRE**T∗**SAMWS	−	+	−	−
Ser-155	RCRET**S∗**AMWSF	−	+	+	−
Thr-177	RRALF**T∗**GGSDP	+	−	−	−
Thr-298	MFLRL**T∗**HPELC	−	+	+	−
Ser-361	VARKH**S∗**ETVLT	−	+	+	+
Thr-409	LLHNV**T∗**VHRRF	−	+	−	−
Thr-416	HRRFH**T∗**DMFHH	−	+	+	−
Thr-689	AFRRT**T∗**SIICE	−	+	+	−
Ser-690	FRRTT**S∗**IICEE	−	+	+	+

a Ser/Thr sites (∗) and surrounding sequence.

b +, known autophosphorylation site.

c ANN, Artificial Neural Network (cut-off = 0.55).

d PSSM, Position-Specific Scoring Matrix (cut-off = 0.80).

e SVM, Support Vector Machine (cut-off =0.55).

Alone or in combination, we mutated all 17 potential binding residues in UL97 to identify those responsible for 14-3-3 association. Substitution of Ser-13 or double substitution of threonines 409 and 416 with nonphosphorylatable alanine residues reduced or eliminated UL97 association (Fig. 4*B*) with all five 14-3-3 proteins tested (β, ε, η, γ, and θ). Substitution of Ser-11 with alanine also reduced UL97 association with 14-3-3 β and γ (Fig. 4*B*). However, the Thr-409/Thr-416 substitution mutant accumulated to lower levels than UL97-WT and did not autophosphorylate (Fig. 4*B*). Residues Thr-409/Thr-416 are not located in the UL97 14-3-3 binding domain (Figs. 3 and 4*A*) but likely render UL97 unable to bind 14-3-3 proteins due to a lack of autophosphorylation (or kinase activity). Therefore, we suspect Thr-409 and Thr-416 are not *bona fide* binding sites on UL97 for 14-3-3 proteins.

Ser-13 (and to a lesser extent Ser-11), however, does appear to be a real 14-3-3 binding site on UL97. UL97-S13A autophosphorylates to some degree as judged by electrophoretic mobility but does accumulate to lower levels than UL97-WT (Fig. 4*B*). However, additional dose-response experiments (Fig. 4*C*) and their quantitation (Fig. 4*D*) showed that UL97-S13A, even when overexpressed and accumulating to levels comparable to UL97-WT, still fails to interact with or shows reduced interaction with all 14-3-3 proteins tested. A double alanine substitution of both Ser-11 and Ser-13 did not substantially decrease 14-3-3 binding to levels lower than UL97-S13A (Fig. 4*B*). Although double phosphomimetic mutants (S11D/S13D or S11E/S13E) did not efficiently interact with 14-3-3 proteins (Fig. 4*B*), other reports with 14-3-3 binding proteins have also failed to recreate 14-3-3 binding of target proteins with phosphomimetic mutations (37, 38), perhaps due to the inexact mimicking that phosphomimetic residues provide. We conclude that Ser-13 of UL97 mediates binding to the 14-3-3 proteins.

### Serine 13 and 14-3-3 function contribute to the protein stability of transfected UL97

**Figure 5:**
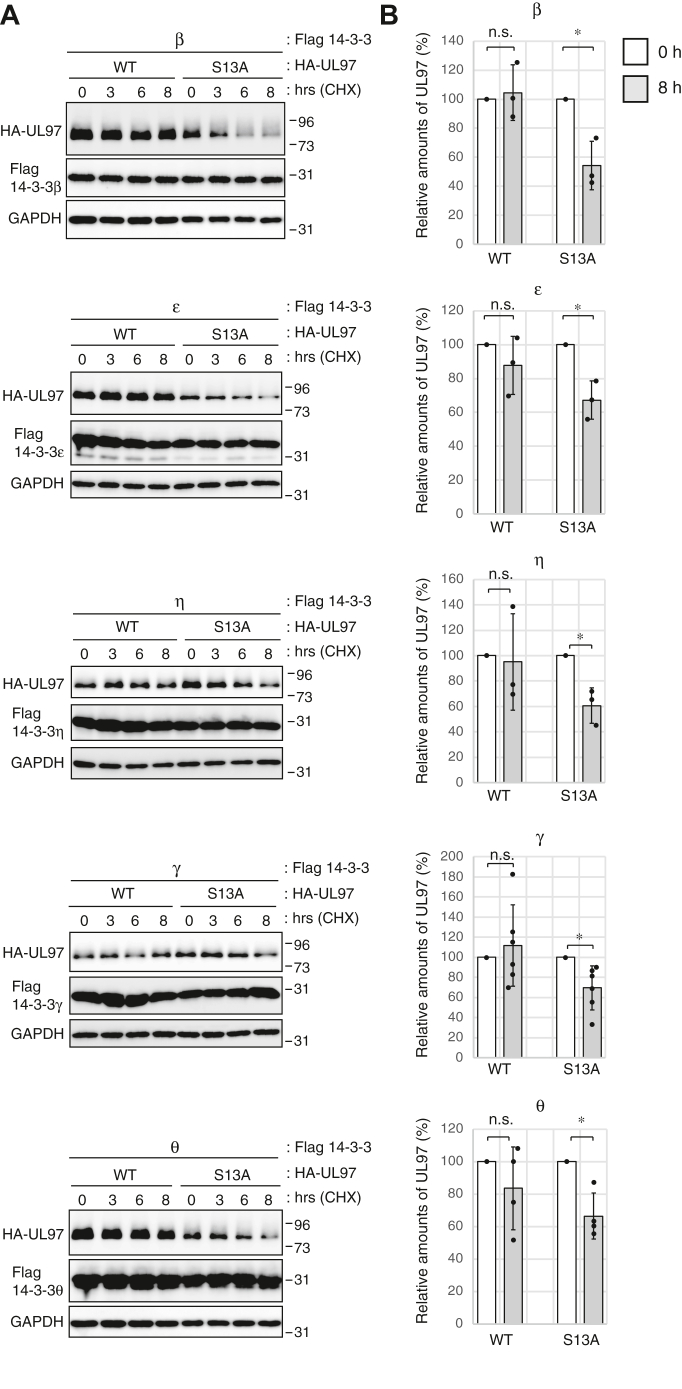
**Serine 13 contributes to the protein stability of transfected UL97.***A*, U-2 OS cells were transfected with an expression vector for WT UL97 (0.6 μg) or the S13A mutant of UL97 (1.2 μg) together with expression vectors for the indicated Flag-tagged 14-3-3 isoform. Plasmid amounts in transfections were equalized with empty vectors. After 48 h, cells were treated with 50 μg/ml of cycloheximide (CHX) for the indicated times, and harvested lysates were subjected to Western blotting with the indicated antibodies. A representative image from one of between three and six independent biological replicates is shown. *B*, the protein level of HA-tagged UL97 from the experiments shown in panel (*A*) was quantified and normalized to GAPDH protein. Values are presented relative to the value in 0 h after CHX treatment of each transfection of WT or S13A-UL97 (set at 100%). Error bars denote SDs. Statistical analysis utilized a two-tailed unpaired Student’s *t* test. ∗*p* < 0.05; n.s., not significant (*p* > 0.05).

14-3-3 proteins promote the stability of their clients (28, 37). In our coIP assays (Fig. 4), the 14-3-3 nonbinding mutant of UL97 (S13A) accumulated to lower levels than WT UL97, prompting us to ask whether the mutant was less stable than the WT protein. WT UL97 coexpressed with each of the 14-3-3 proteins to which it binds (β, ε, η, γ, or θ) showed essentially no decay in protein levels after an 8 h time course treatment with the protein synthesis inhibitor, cycloheximide, as judged both by observing (Fig. 5*A*) and quantitating (Fig. 5*B*) Western blots. In contrast, during coexpression with the 14-3-3 proteins, UL97-S13A levels decreased over time (Fig. 5*A*) showing a significant difference by 8 h (Fig. 5*B*). We conclude the UL97 serine-13 residue that promotes 14-3-3 binding also promotes UL97 stability.

**Figure 6:**
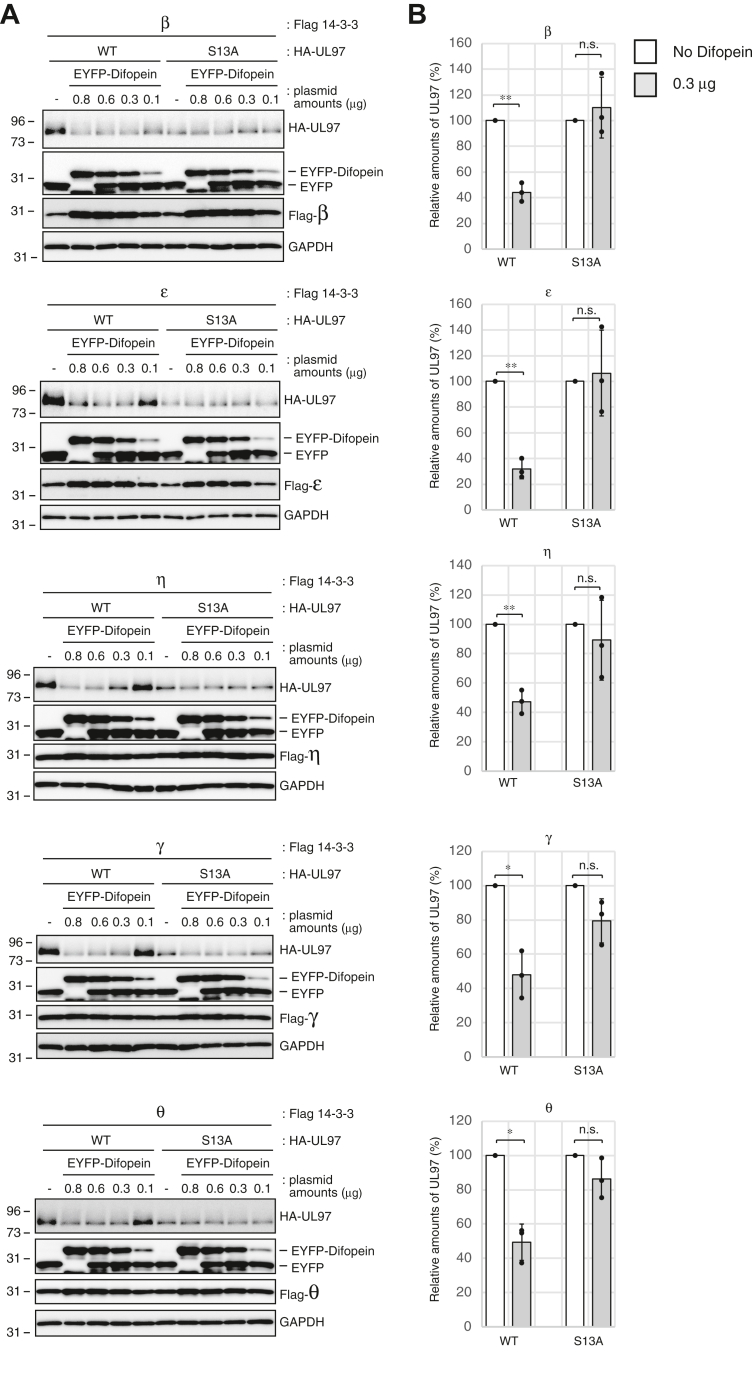
**14-3-3 antagonist Difopein decreases the stability of WT UL97.***A*, U-2 OS cells were transfected with an expression vector for WT or S13A mutant UL97 (0.4 μg) and the indicated Flag-tagged 14-3-3 isoform (0.6 μg) together with the indicated amounts (μg) of an expression plasmid for EYFP fused to Difopein. Plasmid amounts in transfections were equalized with EYFP only expression plasmids. After 48 h, cells were harvested and lysates were subjected to Western blotting with the indicated antibodies. *B*, the transfection with either no (*white bar*) or 0.3 μg (*gray bar*) of an expression plasmid for EYFP fused to Difopein shown in panel (*A*) were performed in biological triplicates. The protein level of HA-tagged UL97 was quantified and normalized to GAPDH. Values are presented relative to the value in the absence of EYFP fused to Difopein for each transfection of WT or S13A-UL97 (set at 100%). Error bars denote SDs. Statistical analysis utilized a two-tailed unpaired Student’s *t* test. ∗*p* < 0.05; ∗∗*p* < 0.01; n.s., not significant (*p* > 0.05).

The S13A mutant that fails to bind 14-3-3 proteins is less stable than WT UL97, implying that 14-3-3 binding may stabilize UL97. To independently test for 14-3-3-mediated stabilization of UL97, we utilized Difopein, a peptide antagonist that disrupts 14-3-3 protein interactions with their clients (38). WT UL97, when coexpressed with each of the 14-3-3 proteins to which it binds (β, ε, η, γ, or θ) and Difopein, accumulated to lower levels than in the absence of Difopein, as judged both by observing (Fig. 6*A*) and quantitating (Fig. 6*B*) Western blots. However, Difopein had no effect on the steady state levels of UL97-S13A (Fig. 6, *A* and *B*) that does not interact with 14-3-3 proteins (Fig. 4). We conclude that 14-3-3 function stabilizes UL97. Combined, we conclude that Ser-13 mediates the binding of 14-3-3 proteins that contribute to the stability of UL97.

### Serine 13 is not required for the kinase activity of transfected UL97

**Figure 7:**
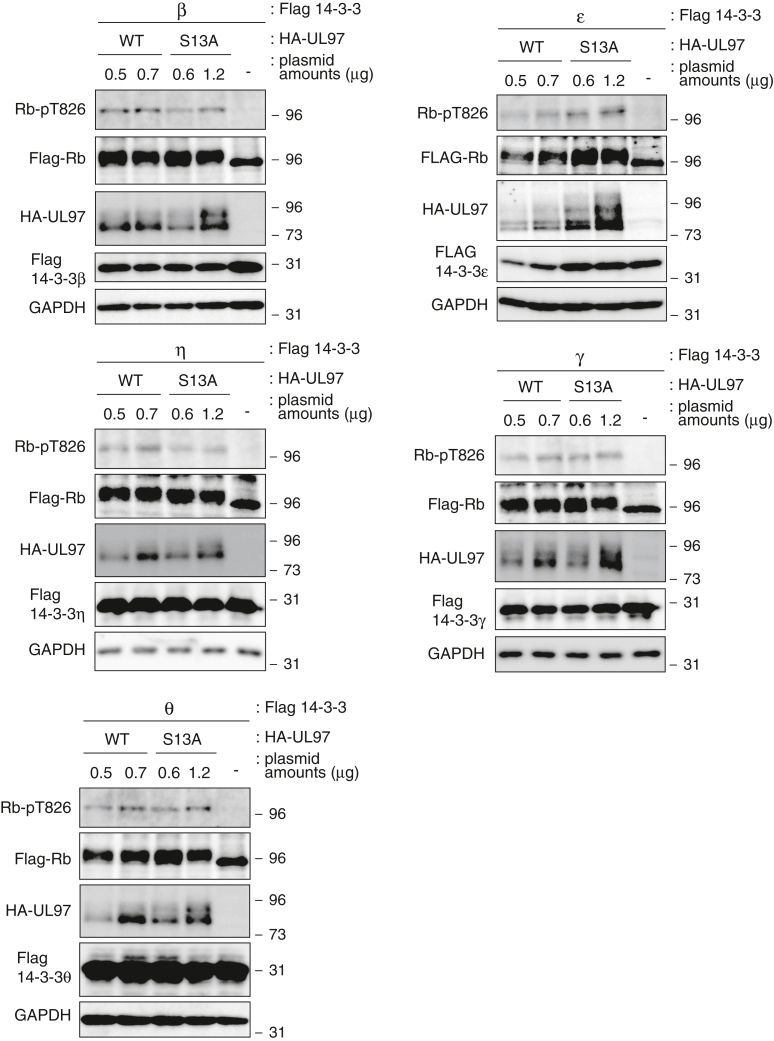
**Serine 13 is not required for the kinase activity of transfected UL97.** Saos-2 cells were transfected for 48 h with the indicated amounts (μg) of an expression vector for WT UL97 or the S13A mutant together with expression vectors for Flag-tagged RB and the indicated Flag-tagged 14-3-3 isoform. Differing amounts of expression vector for WT UL97 or the S13A mutant of UL97 were used. Lysates were analyzed by Western blotting with the indicated antibodies. A representative image from one of three independent biological replicates is shown.

14-3-3 binding can promote protein activity (39). While UL97-S13A showed some ability to autophosphorylate implying it is an active kinase, we wanted to test the ability of this mutant protein to phosphorylate a known UL97 substrate. The retinoblastoma (Rb) tumor suppressor is a well-established substrate for UL97 (1, 2, 4), whose phosphorylation can be detected both by the decreased electrophoretic mobility of the protein on SDS-PAGE, as well as by immunoblotting with a phosphospecific antibody (*e.g*., Rb-P-Thr-826). Rb is not phosphorylated when transfected into Saos-2 cells (Fig. 7) unless a kinase that phosphorylates Rb is cotransfected (40, 41, 42). UL97 phosphorylates Rb during Saos-2 transfections in the absence of overexpressed 14-3-3 proteins (1, 2, 4). However, in an attempt to maximize any differences in UL97-mediated Rb phosphorylation between UL97 and UL97-S13A downstream of differential 14-3-3 binding, we conducted new Saos-2 assays in the presence of overexpressed 14-3-3 proteins. When WT UL97 was coexpressed with each of the 14-3-3 proteins to which it binds (β, ε, η, γ, or θ), coexpressed Rb proteins were phosphorylated (Fig. 7), as expected. To gauge the ability of UL97-S13A to phosphorylate Rb, we transfected slightly larger amounts of plasmid to achieve the steady state protein level of WT UL97, as UL97-S13A is less stable (Fig. 5). Similar to WT, during coexpression with the 14-3-3 proteins, UL97-S13A resulted in Rb phosphorylation (Fig. 7). We conclude that UL97-S13A is an active kinase during transient transfections.

### Phosphorylation of UL97 serine 13 increases its interactions with 14-3-3 proteins

**Figure 8:**
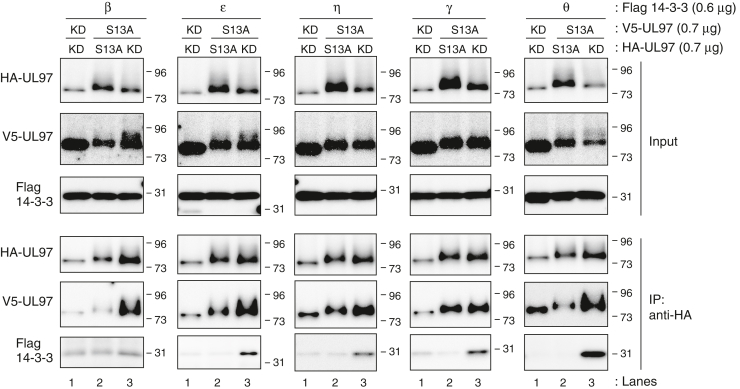
**Phosphorylation of UL97 serine 13 increases its interaction with 14-3-3 proteins.** U-2 OS cells were transfected with an expression vector for either an HA-tagged S13A mutant or a kinase-dead derivative of UL97 (0.7 μg) together with expression vectors for the indicated Flag-tagged 14-3-3 isoform (0.6 μg) and expression vectors for either a V5-tagged S13A mutant or kinase-dead derivative of UL97 (0.7 μg). After 48 h, lysates (Input) were harvested, subjected to immunoprecipitation with anti-HA antibody (IP: anti-HA), and analyzed by Western blot with the indicated antibodies. A representative image from one of two or three independent biological replicates is shown

UL97-KD contains Ser-13 but fails to autophosphorylate and fails to bind 14-3-3 proteins (Fig. 4) implying that Ser-13 phosphorylation is required for binding. Therefore, we trans-autophosphorylated UL97-KD with UL97-S13A to determine if we could drive UL97-KD association with 14-3-3 proteins by phosphorylation. WT UL97 was not used for trans-autophosphorylation because it can both dimerize with the coexpressed UL97-KD as well as bind to 14-3-3 proteins. Therefore, any 14-3-3 proteins coimmunoprecipitating with UL97-KD trans-autophosphorylated by WT UL97 could actually be bound to the WT protein and not UL97-KD. As UL97-S13A does not bind to 14-3-3 proteins (Fig. 4), any 14-3-3 proteins coimmunoprecipitated with UL97-S13A/UL97-KD heterodimers can only be associated with trans-autophosphorylated UL97-KD. Alternatively tagged derivatives of UL97-KD or UL97-S13A were coexpressed with each other and each of the 14-3-3 isoforms to which UL97 binds (β, ε, η, γ, or θ) (Fig. 8). Western blots (Input) visualized UL97-KD trans-autophosphorylation and IP-Western blots detected 14-3-3 proteins bound to trans-autophosphorylated UL97-KD. When HA-UL97-KD was coexpressed with V5-UL97-KD, neither epitope-tagged kinase was phosphorylated and 14-3-3 binding was minimal (lane 1). When HA-UL97-S13A was coexpressed with V5-UL97-S13A, each epitope-tagged kinase was phosphorylated (UL97-S13A is an active kinase (Fig. 7)) but 14-3-3 binding remained minimal (lane 2). However, when HA-UL97-KD was coexpressed with V5-UL97-S13A, each epitope-tagged kinase was phosphorylated and each 14-3-3 protein (excluding perhaps, the β isoform) was efficiently immunoprecipitated with an HA antibody (lane 3), indicating binding to trans-autophosphorylated UL97-KD. We conclude that serine 13 phosphorylation increases the ability of UL97 to interact with 14-3-3 proteins.

### UL97 serine 13 is required for interaction with 14-3-3 proteins during HCMV infection

**Figure 9:**
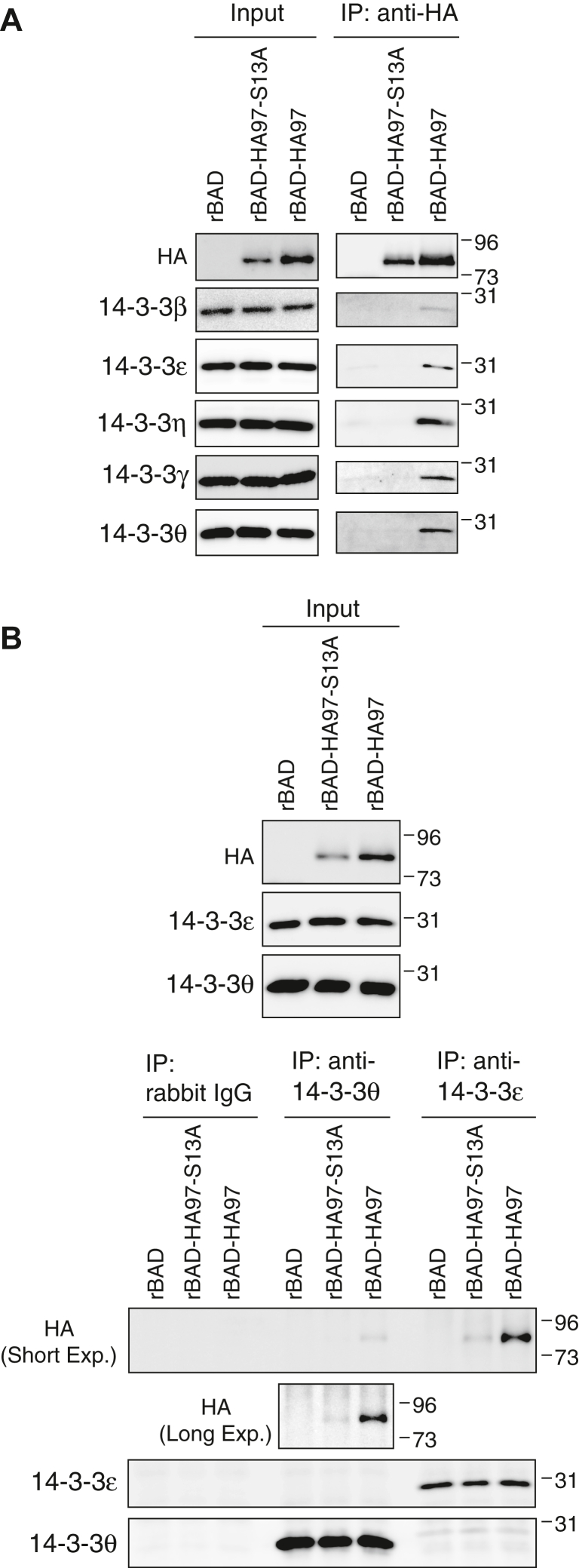
**UL97 Serine-13 is required for interaction with 14-3-3 proteins during HCMV infection.***A*, serum-starved hTERT-BJ1 fibroblasts were infected with the indicated recombinant HCMVs at an MOI of 3. At 4 days post-infection (dpi), lysates (Input) were harvested, subjected to immunoprecipitation with an anti-HA antibody (IP: anti-HA), and analyzed by Western blot with the indicated antibodies. *B*, serum-starved hTERT-BJ1 fibroblasts were infected as in panel (*A*). At 4 dpi, lysates (Input) were harvested, subjected to immunoprecipitation with the indicated 14-3-3 antibody (IP: anti-14-3-3) or rabbit IgG, and analyzed by Western blot with the indicated antibodies. For all panels, representative images from one of three independent biological replicates are shown. MOI, multiplicity of infection.

To determine if UL97 interaction with 14-3-3 proteins had a role during HCMV infection, we generated recombinant HCMVs with amino terminal HA epitope tags on either WT (rBAD-HA97) or S13A (rBAD-HA97-S13A) alleles of UL97 in the AD169 strain of virus. An HA antibody pulled down 14-3-3 β, ε, η, γ, and θ from lysates of hTERT-BJ1 infected with rBAD-HA97 but not rBAD-HA97-S13A (Fig. 9*A*). Reciprocal experiments showed that antibodies to 14-3-3 ε or θ pulled down WT UL97 but not the S13A mutant (Fig. 9*B*). We conclude that UL97 interacts with 14-3-3 proteins during HCMV infection and that Ser-13 is the preferred binding site on UL97 for the 14-3-3 proteins.

### 14-3-3 are relocalized in HCMV-infected cells independently of UL97 association

**Figure 10:**
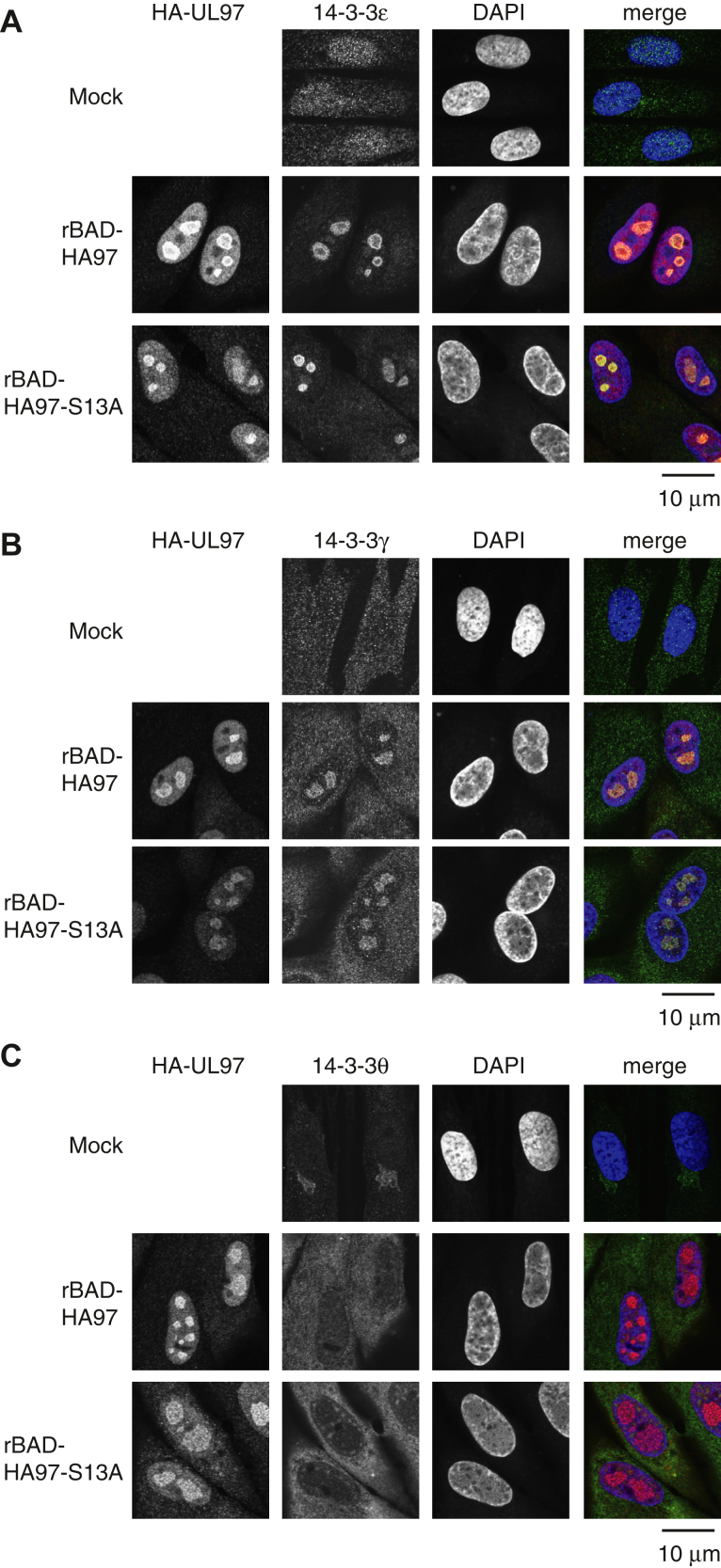
**14-3-3 are relocalized in HCMV-infected cells independently of UL97 association.***A*, serum-starved hTERT-BJ1 fibroblasts were mock infected or infected with the indicated recombinant viruses at an MOI of 3. At 4 days post-infection (dpi), cells were subjected to immunostaining with anti-HA and anti-14-3-3ε antibodies. DNA is stained with DAPI. *B*, indirect immunofluorescence as in panel (*A*) with a 14-3-3γ antibody. *C*, indirect immunofluorescence as in panel (*A*) with a 14-3-3θ antibody. *Blue*: image for DAPI. *Red*: image for anti-HA antibody. *Green*: image for anti-14-3-3 antibody. Experiments were performed in duplicate. DAPI, 4′,6-diamidino-2-phenylindole; MOI, multiplicity of infection.

14-3-3 proteins are known to affect the subcellular localization of their clients (43, 44). To determine if the interaction between UL97 and 14-3-3 proteins affect the subcellular localization of either binding partner, we visualized their localization during HCMV infection. During infection of fibroblasts (Fig. 10), HA-UL97, both WT and the S13A mutant, accumulated in nuclear puncta reminiscent of viral replication compartments where untagged WT UL97 has been found (45). Thus, the inability to bind 14-3-3 does not visibly modify the subcellular localization of UL97 during HCMV productive infection of fibroblasts. Only antibodies to 14-3-3 ε, γ, and θ consistently detected protein by indirect immunofluorescence (IF) in our experiments. In uninfected cells, 14-3-3 ε was dispersed throughout the cytoplasm and nucleus (Fig. 10*A*), 14-3-3γ was dispersed mainly in the cytoplasm (Fig. 10*B*), and 14-3-3θ was peri-nuclear (Fig. 10*C*). During HCMV infection, 14-3-3 ε and γ redistributed to nuclear puncta, presumably viral replication centers, where they colocalized with UL97 (Fig. 10, *A* and *B*). In addition, during HCMV infection 14-3-3θ redistributed to a diffuse cytoplasmic localization distinct from its localization in mock-infected cells (Fig. 10*C*). These 14-3-3 subcellular localization changes occurred similarly during infection with rBAD-HA97 or rBAD-HA97-S13A (Fig. 10), indicating that the ability of UL97 to bind 14-3-3 is not required for the observed subcellular localization changes during infection. We conclude that 14-3-3 ε, γ, and θ relocalize during HCMV infection, independent of UL97 association.

### Serine 13 is not required for the kinase activity of UL97 during HCMV infection

**Figure 11:**
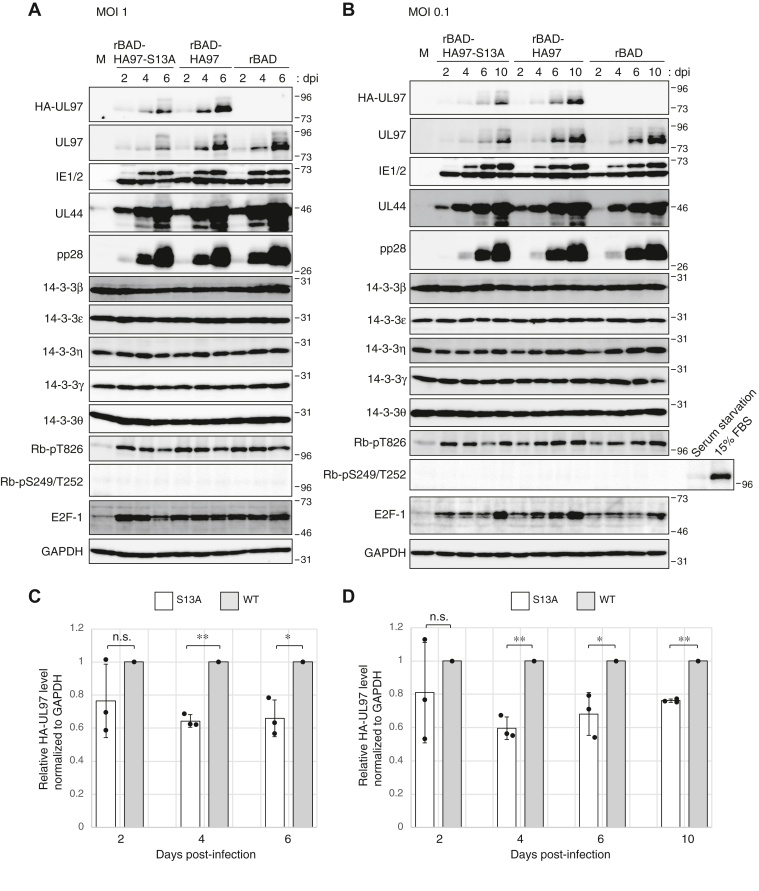
**Serine 13 is not required for the kinase activity of UL97 but contributes to the protein stability of UL97 during HCMV infection.***A*, serum-starved hTERT-BJ1 fibroblasts were mock infected (M) or infected with the indicated recombinant viruses at an MOI of 1. Lysates prepared at the indicated day post-infection (dpi) were analyzed by Western blotting with the indicated antibodies. *B*, analysis as in panel (*A*) except at an MOI of 0.1. For all panels, representative images from one of three independent biological replicates are shown. The mock-infected serum-starved cells were incubated in 0.1% FBS for 48 h (serum starvation) and then cells were stimulated by 15% FBS for 18 h (15% FBS). *C*, the protein levels of HA-tagged UL97 from the experiment shown in panel (*A*) were quantified and normalized to GAPDH levels. The values of HA-tagged UL97-S13A (*white bars*) are presented relative to the values of HA-tagged WT UL97 (*gray bars*: set at 1). Error bars denote SDs from three biological replicates. *D*, analysis as in panel (*C*) except from the experiment in panel (*B*). Statistical analysis utilized a two-tailed unpaired Student’s *t* test. ∗∗*p* < 0.01; ∗*p* < 0.05; n.s., not significant (*p* > 0.05). FBS, fetal bovine serum; MOI, multiplicity of infection.

Because transfected UL97-S13A is an active kinase, we predicted the mutant protein expressed during infection would be active as well. Indeed, the levels of Rb phosphorylated at threonine-826 were similar during HCMV infection with either rBAD-HA97 or rBAD-HA97-S13A at either high (Fig. 11*A*) or low (Fig. 11*B*) multiplicity of infections (MOIs). Neither UL97 nor UL97-S13A phosphorylated the cellular CDK-specific serine-249 or threonine-252 sites (1), as expected (Fig. 11, *A* and *B*). Furthermore, the Rb-suppressed E2F-1 gene product accumulated to similar levels, indicating that Rb was equivalently inactivated by both UL97 and UL97-S13A during infection (Fig. 11). We conclude that UL97-S13A is an active kinase during HCMV infection.

### Serine 13 contributes to the protein stability of UL97 during HCMV infection

Because transfected UL97-S13A is less stable than WT, we predicted the mutant protein would also be less stable during HCMV infection. Indeed, UL97 levels were higher during both high (Fig. 11, *A* and *C*) and low (Fig. 11, *B* and *D*) MOI infections of rBAD-HA97 compared to rBAD-HA97-S13A. We conclude serine-13 promotes UL97 stability during HCMV infection.

### Serine 13 is not required for productive HCMV replication in fibroblasts

**Figure 12:**
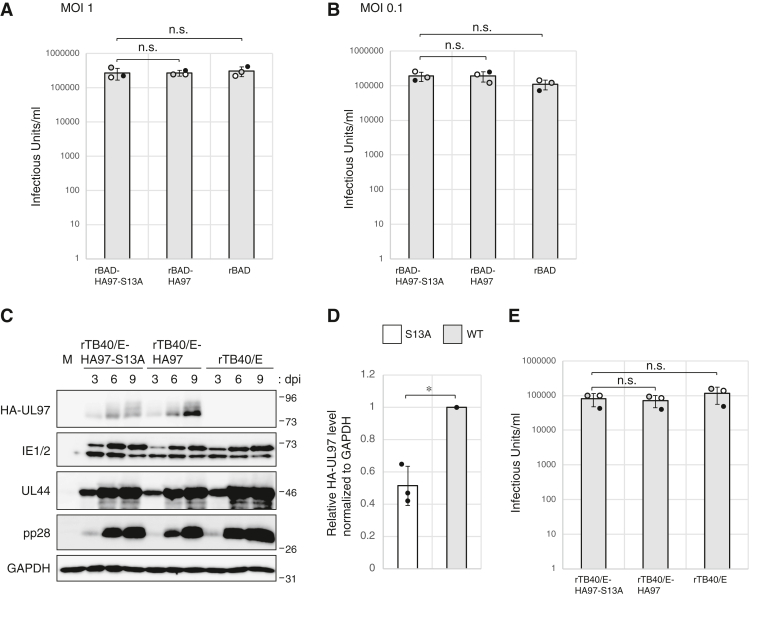
**Serine 13 is not required for productive HCMV replication in fibroblasts.***A*, serum-starved hTERT-BJ1 fibroblasts were infected with the indicated recombinant viruses at an MOI of 1. Cell-free virus was collected at 6 days post-infection (dpi) and titers were determined by plaque assay. Error bars denote SDs from three biological replicates. Statistical analysis utilized a two-tailed unpaired Student’s *t* test. n.s., not significant (*p* > 0.05). *B*, analysis as in panel (*A*) with infections at an MOI of 0.1 and harvest at 10 dpi. *C*, serum-starved hTERT-BJ1 fibroblasts were mock infected (M) or infected with the indicated recombinant viruses at an MOI of 0.1. Lysates prepared at the indicated dpi were analyzed by Western blotting with the indicated antibodies. A representative image from one of three independent biological replicates is shown. *D*, the protein levels of HA-tagged UL97 at 9 dpi from the experiment shown in panel (*C*) were quantified and normalized to GAPDH levels. The value of HA-tagged UL97-S13A (*white bars*) is presented relative to the value of HA-tagged WT UL97 (*gray bars*: set at 1). Error bars denote SDs. Statistical analysis utilized a two-tailed unpaired Student’s *t* test. ∗*p* < 0.05. *E*, cell-free virus was collected at 9 dpi of the experiment shown in panel (*C*) and titers were determined by plaque assay. Error bars denote SDs. Statistical analysis utilized a two-tailed unpaired Student’s *t* test. n.s., not significant (*p* > 0.05). HCMV, human cytomegalovirus; MOI, multiplicity of infection.

Next, we asked if the inability of UL97-S13A to bind 14-3-3 proteins during HCMV infection negatively impacts productive viral replication. During infections at either a high (Fig. 11*A*) or low (Fig. 11*B*) multiplicity, a representative HCMV immediate early (IE1/2), early (UL44), and late protein (pp28) accumulated with indistinguishable kinetics and to indistinguishable levels in fibroblasts infected with either rBAD-HA97 or rBAD-HA97-S13A. Furthermore, rBAD-HA97 and rBAD-HA97-S13A grew to similar titers after high MOI (Fig. 12*A*) or low MOI (Fig. 12*B*). UL97 deficiency generates a more substantial growth defect for the clinical strain TB40/E virus than it does for the laboratory strain AD169 virus used in the previous experiments (46). To test if UL97 association with 14-3-3 proteins is required for efficient replication of the TB40/E strain, we generated recombinant HCMVs with amino terminal HA epitope tags on either WT (rTB40/E-HA97) or S13A (rTB40/E-HA97-S13A) alleles of UL97 in TB40/E. Similar to strain AD169, UL97 levels were higher during infection with rTB40/E-HA97 as compared to rTB40/E-HA97-S13A (Fig. 12, *C* and *D*). However, other viral proteins (IE1/2, UL44, and pp28) accumulated with indistinguishable kinetics and to indistinguishable levels in fibroblasts infected with either rTB40/E-HA97 or rTB40/E-HA97-S13A (Fig. 12*C*). Similar to strain AD169, rTB40/E-HA97 and rTB40/E-HA97-S13A grew to similar titers (Fig. 12*E*). We conclude that 14-3-3 binding by UL97 is dispensable for productive replication of HCMV strains AD169 and TB40/E in fibroblast cells *in vitro*.

**Figure 13:**
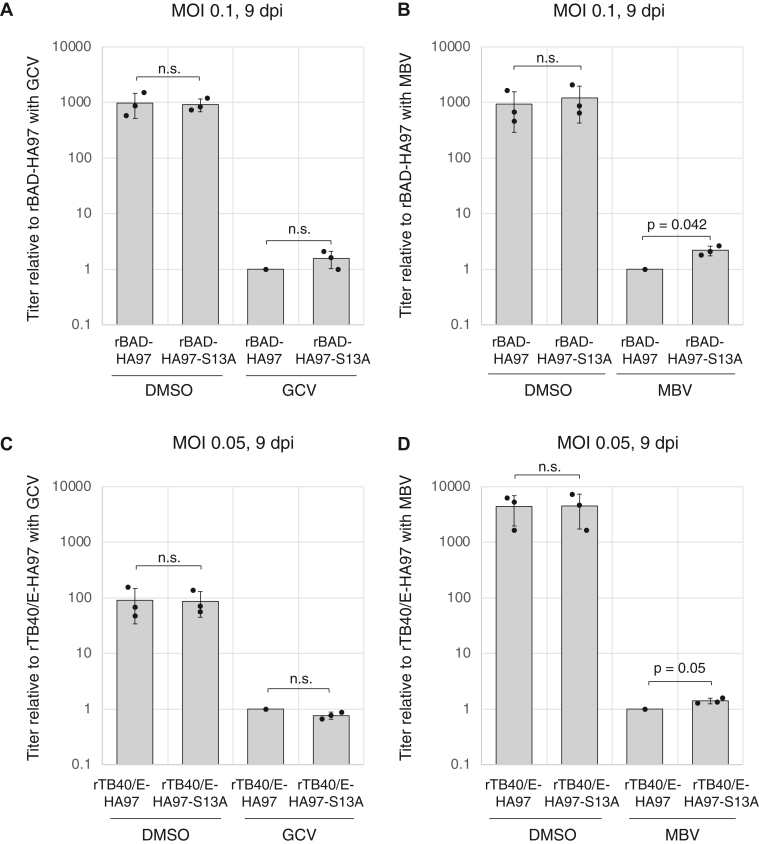
**14-3-3 binding by UL97 minimally effects maribavir sensitivity but not ganciclovir sensitivity.***A*, serum-starved hTERT-BJ1 fibroblasts were infected with the indicated recombinant AD169-based viruses at an MOI of 0.1. Ganciclovir (GCV, 2 μM) or DMSO were added after 1 h. Cell-free virus was collected at 9 days post-infection (dpi) and titers were determined by plaque assay. The value shows virus titer relative to rBAD-HA97 with GCV. *B*, analysis as in panel (*A*) except in the presence or absence of maribavir (MBV, 2 μM). The value shows virus titer relative to rBAD-HA97 with MBV. *C*, serum-starved hTERT-BJ1 fibroblasts were infected with the indicated recombinant TB40/E-based viruses at an MOI of 0.05. GCV (2 μM) or DMSO were added after 1 h. Cell-free virus was collected at 9 days post-infection (dpi) and titers were determined by plaque assay as in panel (*A*). The value shows virus titer relative to rTB40/E-HA97 with GCV. *D*, analysis as in panel (*C*) except in the presence or absence of MBV (2 μM). The value shows virus titer relative to rTB40/E-HA97 with MBV. Error bars denote SDs from three biological replicates. Statistical analysis utilized a two-tailed unpaired Student’s *t* test. n.s., not significant (*p* > 0.05). DMSO, dimethyl sulfoxide; MOI, multiplicity of infection.

Finally, we asked if the inability of UL97-S13A to bind 14-3-3 proteins impacts the ability of UL97 to render infected cells sensitive to the antiviral prodrug ganciclovir (GCV) that it phosphorylates and activates (47, 48) or impacts the sensitivity of UL97 itself to maribavir (MBV), a small molecule inhibitor of UL97 kinase activity (49) recently approved by the FDA for patients with post-transplant CMV infections that do not respond to available antivirals (50, 51). We found that in both AD169 and TB40/E strains, viruses expressing UL97 or UL97-S13A showed similar sensitivity to each drug (Fig. 13, *A–D*), although AD169 and TB40/E expressing UL97-S13A showed subtle resistance to MBV (Fig. 13, *B* and *D*). We conclude that 14-3-3 binding by UL97 minimally affects maribavir sensitivity but not ganciclovir sensitivity. In total, we conclude that serine 13 of UL97 mediates binding to 14-3-3 proteins and promotes the steady state accumulation of the v-CDK, UL97.

## Discussion

Here, we identify and present the novel interaction between HCMV UL97 and cellular 14-3-3 isoforms β, ε, η, γ, and θ. Three 14-3-3 proteins (β, η, and γ) were found in both biological replicates of our UL97 interactome screen. Two additional 14-3-3 proteins (ε and θ) were also found in both biological replicates but also at low level in one of the two controls. However, they showed a >5-fold enrichment over controls (see Supporting information). Similarly, two previously identified UL97-binding partners (HSP90AA1 and TUBA1B) also showed a >5-fold enrichment over controls, even though they were found at low levels in one of the two controls (see Supporting information). The interactions between UL97 and 14-3-3 proteins depend upon both the kinase activity of UL97 as well as the autophosphorylation site at residue serine-13.

The association of 14-3-3 proteins with the N terminus of UL97 enhanced UL97 protein stability. A stability determinant in the N terminus of UL97 matches well with the observation that the full-length protein is the most abundant UL97 isoform in HCMV-infected cells (22). Indeed, the unique long N-terminal region of UL97 is predicted to be structurally disordered (18) and such intrinsically disordered proteins are susceptible to proteolysis (52, 53). The lack of an N-terminal extension in gammaherpesvirus v-CDKs implies that they may not bind 14-3-3 proteins, and indeed our preliminary efforts to isolate complexes between 14-3-3 proteins and BGLF4, the EBV v-CDK, have failed. 14-3-3 proteins regulate protein stability in multiple ways, including blocking proteolysis by sterically preventing client polyubiquitination (28), relocalizing clients away from degradation machinery (54), or can even act as chaperones to help proteins achieve or maintain more stable structures (55). How the 14-3-3 proteins stabilize UL97 remains to be explored.

While we have shown that 14-3-3 proteins stabilize UL97, we have not assayed for any potential effects of UL97 on 14-3-3 function. Interestingly, HCMV UL97 is not the only herpesvirus protein that associates with 14-3-3 proteins. Indeed, the HCMV UL43 and UL48 proteins interact with 14-3-3 proteins (33, 56), as do EBV BPLF1 and KSHV ORF64 (33) orthologs of HCMV UL48. Interestingly, UL97, UL43, and UL48 are all tegument proteins, and 14-3-3 proteins have been identified as virion components of HCMV (57) as well as other herpesviruses (57, 58, 59, 60). It will be interesting to determine if HCMV nonstructural proteins also associate with 14-3-3 proteins, as they do in other viruses (61). As siRNA-mediated knockdown of 14-3-3 proteins inhibits the productive replication of HSV-1 (62), it might be interesting to explore 14-3-3 inhibitors including Difopein (63) as pan-herpesvirus or even broad spectrum viral inhibitors.

Finally, in addition to the 14-3-3 proteins, our UL97 interactome analysis identified 27 potential new UL97 binding partners and/or substrates, as well as 19 previously identified binding partners and/or substrates. The list of new and old targets includes multiple heat shock proteins, as well as DnaJ and TRiC/CCT family chaperones, providing a wealth of opportunities for further exploring the role of UL97 during HCMV infection.

## Experimental procedures

### Cells

U-2 OS, Saos-2, HEK-293T, HFF2/T cells (human foreskin fibroblasts immortalized with human telomerase reverse-transcriptase [hTERT]) (64), and hTERT-BJ1 (hTERT-immortalized human fibroblasts) were grown and maintained at 37 °C in Dulbecco’s modified Eagle’s medium (DMEM, Nacalai) supplemented with 10% fetal bovine serum (FBS, HyClone), 100 units/ml penicillin, 100 μg/ml streptomycin, and 0.292 mg/ml glutamine (PSG, Sigma–Aldrich). For serum starvation, hTERT-BJ1 and HFF2/T cells were incubated in DMEM with 0.1% FBS for 48 h.

### Viruses and Bac mutagenesis

The bacterial artificial chromosome (BAC)–derived recombinant HCMVs were strain AD169 (pAD/Cre) (65) and strain TB40/E (TB40-BAC4) (66). All BAC mutagenesis was conducted using a two-step Red recombination procedure (67). We generated an HCMV mutant, rBAD-HA97-S13A, that expresses UL97 bearing an HA-tag at the N terminus and an alanine substitution of Ser-13 (pAD/Cre-HA97-S13A). The revertant HCMV, rBAD-HA97, that expresses WT UL97 bearing an HA-tag at the N terminus was prepared by BAC mutagenesis with pAD/Cre-HA97-S13A. Recombinant TB40/E HCMVs, rTB40/E-HA97, and rTB40/E-HA97-S13A were generated in the same way. DNA fragments for homologous recombinations were obtained as gBlocks gene fragments from Integrated DNA Technologies (IDT). Sequences are available upon request. Recombinant BAC genomes were verified by sequencing the UL97 gene and flanking regions, as well as by genomic DNA digestion patterns with Xba I, which was introduced at the HA-tag site. Virus titers were determined by plaque assays on hTERT-BJ1 cells cultured in media with 5% FBS. HCMV infections were performed under serum-starved conditions if not otherwise specified.

### Reagents

Maribavir was obtained from Med Chem Express (catalog no.: # HY-16305). Ganciclovir was obtained from Merck (SML2346). Both reagents were dissolved in dimethyl sulfoxide and added into the infected cells at a final concentration of 2 μM. Cycloheximide was obtained from Sigma–Aldrich and added to media at a final concentration of 50 μg/ml.

### Plasmids

Expression plasmids have been described for the following: pCGN-based HA-tagged UL97 and kinase-deficient derivative (2); pFC14a-HA-UL97 WT (5); pcDNA3-based V5-tagged kinase-dead UL97 (68); pCGN71 for reconstitution of the recombinant viruses (69); and pEYFP-C1-Difopein (38). pcDNA3-based Flag-tagged 14-3-3 β, ε, η, γ, σ, θ, and ζ were constructed by inserting PCR products amplified from pGEX-6P-1 carrying each 14-3-3 protein (70) into BamHI and NotI sites in pcDNA3 (Invitrogen) using In-Fusion HD cloning kit (Clontech). pCGN-based HA-tagged UL97 fragments (N1, 1–278 aa; C1, 279 to 707 aa; N2, 1 to 486 aa; C2, 487 to 707 aa) were constructed by inserting PCR products amplified from pCGN-HA-UL97 (full length 1–707 aa) into XbaI and BamHI sites in pCGN using In-Fusion HD cloning kit. The following expression plasmids were generated during this study by site-directed mutagenesis techniques: pCGN-based HA-tagged UL97-S2/3A, S11A, S13A, S11/13A, S11/13D, S11/13E, T16/18A, S133A/T134A, T154A/S155A, T177A, T298A, S361A, T409/416A, and T689A/S690A; pcDNA3-based V5-tagged UL97-S13A; pcDNA3-based Flag-tagged 14-3-3β (S147A/Y180H/C191I), ε (S148A/Y181H/C192I), η (S150A/Y183H/C194I), γ (S150A/Y183H/C194I), σ (A147S/H180Y/I191C), θ (S145A/Y178H/C189I), θ (C134A), and ζ (A134C). All alleles generated by PCR were confirmed by sequencing. The sequences of primers for mutagenesis are available upon request.

### Western blotting

HCMV-infected fibroblasts or Rb-transfected Saos-2 cells were lysed in an SDS solution (1% SDS, 2% β-mercaptoethanol) by boiling for 10 min, followed by vortexing as previously described (1). Images were obtained with either LAS-4000 mini and Image Reader LAS-4000 software (Fujifilm) or LuminoGraph I and ImageSaver6 software (ATTO). Quantification of bands was performed with Multi Gauge v3.1 software (Fujifilm) or CS Analyzer 4 software (ATTO), respectively. Statistical analyses utilized a two-tailed unpaired Student’s *t* test.

### IPs

U-2 OS cells (2 × 10^5^/35 mm plate) were transfected with pcDNA3-Flag-14-3-3 (1 μg) together with pCGN vector carrying WT/mutated HCMV UL97 (0.6 μg) using TransIT-2020 (Mirus). After 48 h, cells were suspended with IP buffer (50 mM Tris, pH7.5; 0.1 M NaCl; 1% NP40; 10 μg/ml pepstatin A; 25 μg/ml leupeptin; 1 mM PMSF; 25 mM NaF; 10 mM β-glycerophosphate) as described previously (70). The extracts were immunoprecipitated with anti-HA mAb-magnetic beads (MBL, M180-11). Beads were washed three times with NET gel buffer (150 mM NaCl; 50 mM Tris, pH7.4; 0.1% Triton X-100; 1 mM EDTA) and eluted with SDS gel loading buffer. For the IP assays of HCMV-infected cells, cells were lysed as described previously. Extracts were incubated with anti-14-3-3ε, 14-3-3θ or UL97 antibodies, or normal rabbit IgG as control for 5 h and then incubated with protein A/G-magnetic beads (Pierce) for 2 h or directly incubated with anti-HA mAb-magnetic beads for 5 h. Beads were washed and eluted as described previously.

### IF analysis

HCMV-infected hTERT-BJ1 cells were fixed with 2% paraformaldehyde and stained as previously described (5) with anti-HA and anti-14-3-3ε, γ, θ antibodies and with secondary antibodies conjugated to Alexa Fluor 488 or 594 (Molecular Probes). Samples were mounted with 4′,6-diamidino-2-phenylindole. The images were captured using Carl Zeiss LSM710 confocal microscope and software (ZEN2009).

### Antibodies

Primary antibodies were purchased from Abcam (14-3-3β, catalog number ab15260 for Western blotting [WB]; 14-3-3η, ab206292 for WB; Rb phospho-Thr-826, ab133446), Ambion (GAPDH, AM4300), Bethyl laboratories (14-3-3θ, A303-146A for WB, IF, and IP), Cell Signaling Technology (E2F-1, 3742), Covance (HA, MMS-101P); GeneTex (14-3-3ε, GTX109090 for WB, IF, and IP; 14-3-3γ, GTX113298 for WB and IF), Invitrogen (14-3-3η, PA5-81128 for WB; V5, R960-25; Rb phospho-Ser-249/phospho-Thr-252, 44-584G), MBL (GFP, M048-3), Millipore (HCMV IE1/IE2, MAB8131), Sigma–Aldrich (FLAG-M2, F1804), and Virusys (HCMV pp28, CA004-100; UL44, CA006-100). Antibodies against HCMV UL97 have been previously described (11).

### Halo tag pull downs and LC-MS/MS analysis

Kinase was purified from HEK-293T cells transfected with pFC14a-HA-UL97 WT or pFC14a (Promega) with a 1 h bead incubation as previously described (5). Samples were eluted with TEV protease (Promega) and submitted to the University of Wisconsin Biotechnology Center for LC-MS/MS analysis with a Thermo Fisher Scientific Orbitrap Elite. Two independent biological replicates were analyzed. Results were analyzed using Mascot and Scaffold viewer, and peptides proteins reported had at least one identifying peptide with 1% false discovery rate threshold. In Table 1, the listed proteins were detected in HA-UL97-expressed samples but not in controls (pFC14a) and ordered by score (quantitative value normalized total spectra).

## Data availability

The data supporting the findings of this study are available within the article and its supporting information.

## Supporting information

This article contains supporting information.

## Conflict of interest

The authors declare that they have no conflicts of interest with the contents of this article.
